# Development of a Risk Score Model for Osteosarcoma Based on DNA Methylation-Driven Differentially Expressed Genes

**DOI:** 10.1155/2022/7596122

**Published:** 2022-05-13

**Authors:** Yuxiang Kang, Guowang Li, Guohua Wang, Zhenxin Huo, Xiangling Feng, Lilong Du, Yongjin Li, Qiang Yang, Xinlong Ma, Bingbing Yu, Baoshan Xu

**Affiliations:** ^1^Department of Minimally Invasive Spine Surgery, Tianjin Hospital, Tianjin, China; ^2^Graduate School of Tianjin Medical University, Tianjin, China; ^3^Department of Pharmacology, Tianjin Key Laboratory of Inflammation Biology, School of Basic Medical Sciences, Tianjin Medical University, Tianjin, China; ^4^Department of Orthopedics, The Fifth Affiliated Hospital of Guangzhou Medical University, Guangzhou, China; ^5^Key Laboratory of Biological Targeting Diagnosis, Therapy and Rehabilitation of Guangdong Higher Education Institutes, The Fifth Affiliated Hospital of Guangzhou Medical University, Guangzhou, China

## Abstract

Osteosarcoma (OS) is the commonest malignant bone tumor in adolescent patients, and patients face amputation, tumor metastasis, chemotherapy resistance, and even death. We investigated the potential connection between abnormal methylation differentially expressed genes and the survival rate of osteosarcoma patients. GSE36002 and GSE12865 datasets of GEO database were utilized for abnormal methylation differentially expressed genes, followed by function and pathway enrichment analyses, the protein-protein interaction network in the STRING database, and cluster analysis in the MCODE app of Cytoscape. The RNA-seq and clinical data from the TARGET-OS project of TCGA were used for univariate and least absolute shrinkage and selection operator (LASSO) Cox regression analyses to predict the risk genes of osteosarcoma. 1191 hypermethylation-downregulated genes might function through plasma membrane, negative regulation of transcription from the RNA polymerase II promoter, and pathways, including transcriptional misregulation in cancer. 127 hypomethylation-upregulated genes were enriched in proteolysis, negative regulation of the canonical Wnt signaling pathway, and metabolic signaling pathways. The univariate Cox analysis revealed 638 genes (*P* < 0.01), including 50 hypermethylation-downregulated genes and 4 hypomethylation-upregulated genes, subsequently based on LASSO Cox regression analysis for 54 aberrant methylation-driven genes, and three genes (COL13A1, MXI1, and TBRG1) were selected to construct the risk score model. The three genes (COL13A1, MXI1, and TBRG1) regulated by DNA methylation were identified to relate with the outcomes of OS patients, which might provide a new insight to the pathological mechanism of osteosarcoma.

## 1. Introduction

Osteosarcoma is the most common primary bone malignancy. The annual incidence of new osteosarcoma, confirmed with histopathology, is 1.8 per million in Finland [1], and approximate 800 new osteosarcoma patients are diagnosed each year in the United States, 400 of whom are under the age of 20 [2].

Currently, the overall 5-year survival rate for primary osteosarcoma is 68% and the survival rate of patients with metastasis or recurrences is less than 20%. The survival rate improved from 11% with surgical resections in the 1960s to 70% with combined chemotherapy in the mid-1980s. For the past 40 years, surgical techniques have made significant progress, but a considerable number of patients still face the threat of amputation and the survival rate has not made any progress [3]. Therapies for preventing tumor metastasis and chemotherapy resistance are urgently needed [4].

Epigenetics is defined as changes in gene expression levels in the absence of changes in gene sequence, including DNA methylation, histone modifications, and noncoding RNAs [5]. Understanding the epigenetic mechanisms of osteosarcoma may give us an insight into its pathophysiological characteristics [6]. DNA methylation is significant for physiological phenomena, such as regulating transcription, chromosome structure, and genome stability [7]. It mainly occurs in the CpG island region and suppresses gene expression by compacting and inactivating the chromatin structure, making the transcription complex inaccessible [8]. Abnormal methylation has severe effects on gene expression in a variety of tumors [9, 10].

Recently, genomic microarrays and RNA-seq can be used to detect epigenetic alternations in the pathogenesis of the tumor. Here, we applied the GSE36002 and GSE12865 datasets of the GEO database for expression profiles of hypomethylation-upregulated genes (HUGs) and hypermethylation-downregulated genes (HDGs) in osteosarcoma, and subsequently, the annotated function and pathway, protein-protein interaction (PPI) network, and cluster analysis was performed. The RNA-seq and clinical data from the TARGET-OS project in The Cancer Genome Atlas (TCGA) program were employed for univariate and LASSO Cox regression analyses. The correlation between survival rate and aberrant methylation differentially expressed genes was explored. We aim to elucidate the effect of DNA methylation on the development of osteosarcoma and thus provide new therapeutic targets for osteosarcoma.

## 2. Materials and Methods

### 2.1. Microarray Datasets and Processing

The gene expression dataset (GSE12865) and the gene methylation dataset (GSE36002) were downloaded from Gene Expression Omnibus (GEO, https://www.ncbi.nlm.nih.gov/Geo/) of the National Center for Biotechnology Information (NCBI). The gene expression dataset GSE12865 included 12 samples from pediatric osteosarcoma and 2 samples of healthy osteoblast cells on the basis of the platform of GPL6244 (Affymetrix Human Gene 1.0 ST Array). The gene methylation dataset GSE36002 included 19 samples from osteosarcoma cell lines and 6 samples from healthy people based on the platform of GPL8490 (Illumina HumanMethylation27 BeadChip). We utilized GEO2R to save all the differently expressed genes and abnormally methylated genes in GSE12865 and GSE36002, with *P* < 0.05 and |t| > 2 as the inclusion standards with after Student's *t* test [11]. HDGs and HUGs were determined with the Venn (http://bioinformatics.psb.ugent.be/webtools/Venn/).

### 2.2. Function and Pathway Enrichment Analysis

Function and pathway enrichment analyses of the abnormal methylation differentially expressed genes were implemented through DAVID database (https://david.ncifcrf.gov/).

### 2.3. Protein-Protein Network Construction

We separately constructed the PPI network of HDGs and HUGs with the STRING database (https://string-db.org/). The minimum required interactive score of high confidence (0.9) was considered a cutoff criterion, and the active interaction source was limited to text mining, experiments, and databases.

### 2.4. Cluster Analysis and Key Gene Screening of the PPI Network

CytoHubba app was used to identify key genes of HDGs and HUGs by the Matthews correlation coefficient (MCC) algorithm with node = 5 as criterion in Cytoscape. Cluster analysis of HDGs and HUGs was performed with the Molecular Complex Detection (MCODE) app, with degree cut off = 2, node score cut off = 0.2, k − score = 2, and max depth = 100 regarded as significant.

### 2.5. Screening of Prognostic Factors

The Therapeutic Application Research to Generate Effective Treatments (TARGET) project was aimed at identifying molecular changes that lead to childhood cancer, and the main purpose of the project is to use open source databases to document some genetic and clinical information that will help develop novel, effective, and less toxic therapy. The TARGET-OS project includes RNA-seq data and associated clinical information of 88 osteosarcoma patients at http://portal.gdc.cancer.gov/. We downloaded TARGET-OS RNA sequencing data through R studio and merged the mRNA expression of genes with clinical information (vital status and survival time), and there were 3 patients whose sequencing data and clinical information did not match. Subsequently, we used univariate Cox regression to screen for genes associated with survival (*P* < 0.01).

### 2.6. Risk Scoring Model Construction

First, we performed an overlap analysis of survival-related genes and aberrant methylation-driven genes. Second, we apply LASSO Cox regression to further narrow down, using the glmnet package in R. LASSO regression is a method that uses the L1 penalty to shrink the regression coefficients to zero. This approach also reduces dimensionality and avoids collinearity between variables. Finally, Using X-tile, a bioinformatic tool for biomarker assessment and outcome-based cut-point optimization [12], to determine appropriate cutoff values, we divided 85 osteosarcoma patients in TARGET-OS into low-risk and high-risk groups. ROC curves and the survival ROC package in R were used to verify the predictive power of risk models.

### 2.7. Statistical Analysis

R (version 4.0.0, http://www.r-project.org/) was used for statistical analysis. *P* < 0.05 was considered statistically significant. We performed all analyses following the relevant guidelines in R.

## 3. Results

### 3.1. Identification of HDGs and HUGs in OS

**Figure 1:**
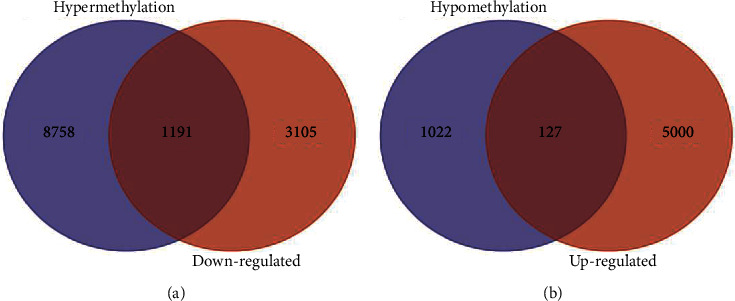
Venn map of gene methylation dataset (GSE36002) and gene expression dataset (GSE12865): (a) hypermethylation-downregulated genes and (b) hypomethylation-upregulated of genes.

There were 5127 upregulated genes and 4296 downregulated genes in GSE12865 (P < 0.05, |t| > 2). There were 9949 hypermethylated genes, and 1149 hypomethylated genes in GSE36002 (P < 0.05, |t| > 2). 1191 HDGs were obtained by combining 9949 hypermethylated genes and 4296 downregulated genes. 127 HUGs were obtained by combining 1149 hypomethylated genes and 5127 upregulated genes (Figure 1).

### 3.2. GO and KEGG Enrichment Analysis of HDGs and HUGs

**Figure 2:**
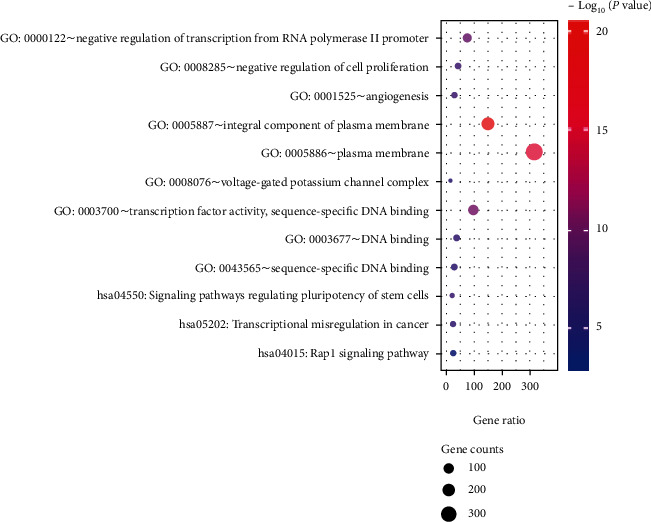
Function and pathway enrichment analysis of hypermethylation-downregulated genes.

As presented in Figure 2, HDGs were enriched in the plasma membrane and voltage-gated potassium channel complex in the aspect of cell component. In the biological process category, HDGs were primarily enriched in the negative regulation of transcription from the RNA polymerase II promoter and anterior/posterior pattern specification, and in the molecular function, HDGs were mainly enriched in transcription factor activity and sequence-specific DNA binding, and the enriched pathways were transcriptional misregulation in cancer and pathways in cancer.

**Figure 3:**
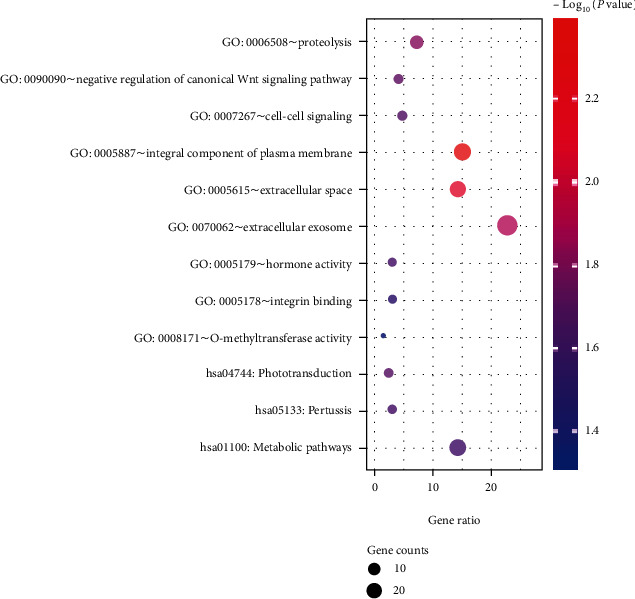
Function and pathway enrichment analysis of hypomethylation-upregulated genes.

As shown in Figure 3, in the cellular components, HUGs were enriched in the integral component of the plasma membrane, extracellular exosome, and extracellular region. In biological processes, HUGs were mainly enriched in proteolysis and negative regulation of the canonical Wnt signaling pathway. In molecular function, the enrichment was in hormone activity, integrin binding, and O-methyltransferase activity. The enriched pathways of the HUGs were metabolic pathways and phototransduction.

### 3.3. PPI Network Construction and Module Analysis of HDGs and HUGs

**Figure 4:**
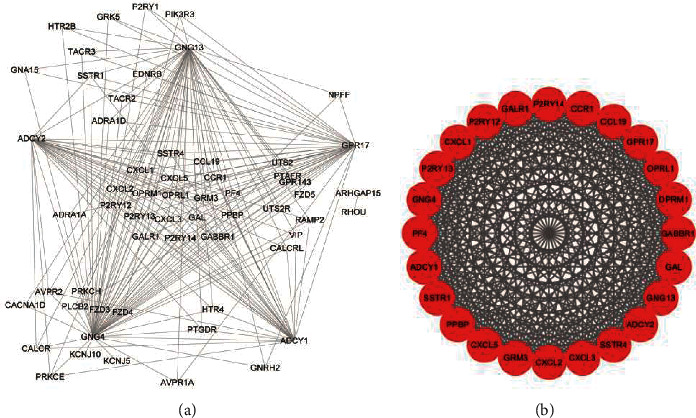
PPI network and top1cluster of hypermethylation-downregulated genes: (a) PPI network of top 5 genes and neighborhoods in hypermethylation-downregulated genes and (b) top1 cluster.

**Figure 5:**
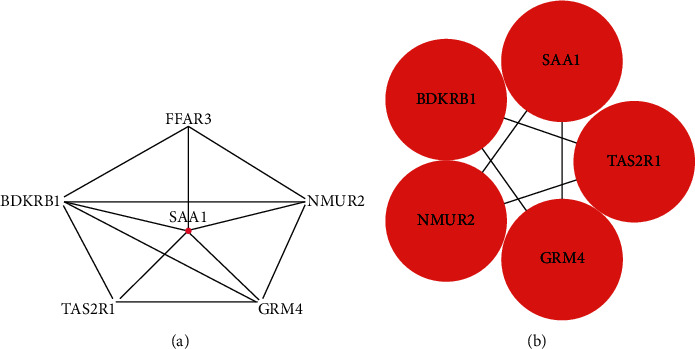
PPI network and top1 cluster of hypomethylation-upregulated genes: (a) PPI network of top 5 genes and neighborhoods in hypomethylation-upregulated genes and (b) top1 cluster.

The PPI network of the top 5 HDGs and neighborhoods was represent in Figure 4(a), and the top1 cluster was revealed in Figure 4(b); the PPI network of the top 5 HUG and neighborhoods was shown in Figure 5(a), and the top1 cluster was demonstrated in Figure 5(b).

### 3.4. Development of the Risk Score Model for OS

**Figure 6:**
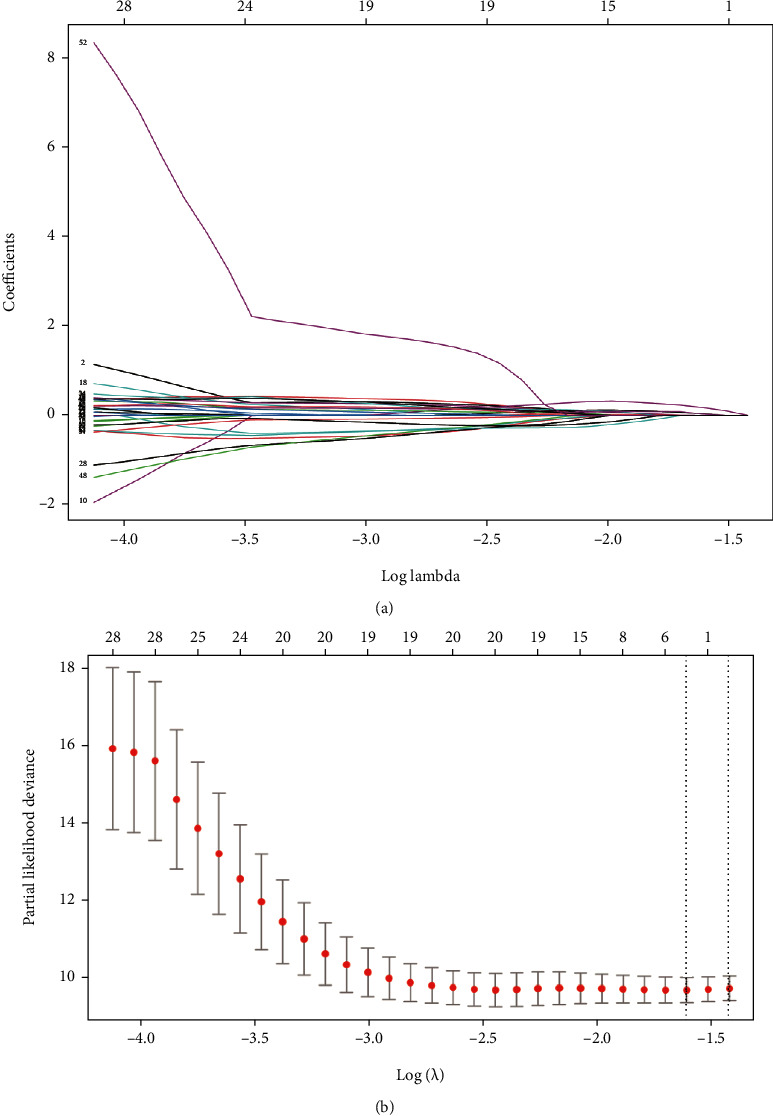
LASSO regression analysis of methylation-driven genes: (a) LASSO coefficients and (b) Plots of the tenfold cross-validation error rates. The dotted lines indicate the minimal standard error and the optimal *λ* value.

During the development of the operating system risk scoring model, first, we used univariate Cox regression to explore the relationship between gene expression and survival status and survival time of the TARGET-OS project. 638 genes were analyzed as potential genes significantly associated with survival status and time (*P* < 0.01), and the top ten genes are shown in Figure S1A. Then, Venn analysis of 1318 aberrant methylation-driven genes and 638 OS-related genes is shown in Figure S1B, 54 genes were selected for 1000 repetitions of LASSO regression analysis, and 10-fold cross-validation was used to select 3 genes with non-Zero coefficients to serve as seed genes (Figures 6(a) and 6(b)), and finally screened by risk score model for 3 genes (COL13A1, MXI1, and TBRG1):Risk score = (0.0339348∗COL13A1 mRNA level) + (0.1748218∗MXI1 mRNA level) + (0.0303896∗TBRG1 mRNA level).

**Figure 7:**
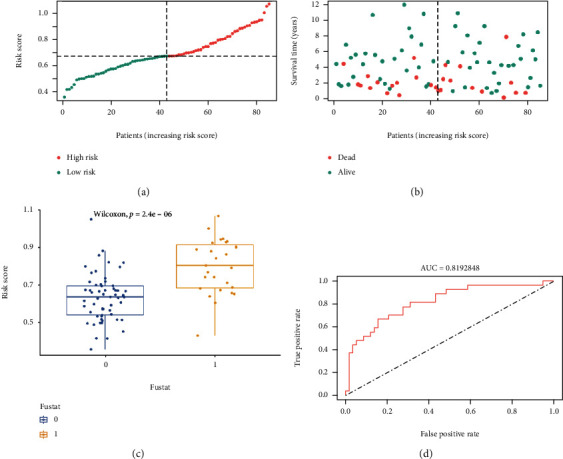
Risk scoring model. (a) Distribution of risk scores. The red and green dots represent the high-risk group and the low-risk group, respectively. (b) Survival distribution for high-risk or low-risk score groups. The red and green dots represent the dead and the live, respectively. (c) Distribution of risk scores for different survival states. 0 and 1 represent the live and the dead, respectively. (d) ROC curve of the risk scoring model.

We calculated a risk score for each patient and then divided the patients into low- and high-risk groups by X-tile. The high-risk group had more deaths than the low-risk group (Figures 7(a)a and 7(b)). The distribution and Wilcoxon test of risk scores of patients with different survival status in the TCGA database were shown in Figure 7(c). The ROC curve of the risk score model developed by the three genes driven by abnormal methylation (COL13A1, MXI1, and TBRG1) was shown in Figure 7(d), with an AUC of 0.819.

## 4. Discussion

Osteosarcoma is a major challenge and ranks first in primary bone tumors [13]. Previous studies have demonstrated that tumorigenesis may be related to whether aberrant methylation status drives gene silencing of oncogenes and tumor suppressor genes [14]. Evidences have emerged revealing that osteosarcoma is a differentiated disease caused by epigenetic changes that interferes the differentiation of stem cells into osteoblasts [15]. Exploring DNA methylation in osteosarcoma may illuminate the mechanisms of disease development.

As was demonstrated, HDGs were enriched in regulation of transcription from the RNA polymerase II promoter, transcription factor activity and sequence-specific DNA binding, plasma membrane, voltage-gated potassium channel complex, the signaling pathways regulating the pluripotency of stem cells, and transcriptional misregulation in cancer and Rap1 signaling pathway. There is no doubt that transcriptional misregulation and pathways in cancer are involved in osteosarcoma.

In plasma membrane proteomic analysis that compared human osteosarcoma and normal osteoblasts, cell membrane components accounted for 69% of the differentially expressed proteins, which were involved in cell adhesion, signal transduction, cellular structure, and biological processes involved in cell-cell contact [16]. Dysregulation of these highly dynamic membrane domains can promote oncogenic signaling [17].

The RNA polymerase II core promoter is simply labeled as a sequence that directs transcription initiation; however, it can mediate many complex transcriptional patterns and responses to enhancers [18]. Evidences have suggested that various sequence-specific transcription factors and transcriptional enhancers have specific effects on the core promoter, and promoter hypermethylation can perform transcriptional silencing of tumor suppressor genes in cancer. Inactivation of tumor suppressor function is found in osteosarcoma and acts as a key role in its pathogenesis [19]. Hypermethylation of the promoter located in the CpG island could influence genes of apoptosis, cell cycle, carcinogen metabolism, DNA repair, angiogenesis, and cell-cell interaction, all of which contribute to cancer development [14].

Ras-related protein-1 (Rap1) signaling pathway regulates cell adhesion and influences the expression of matrix metalloproteinases (MMPs), thus taking effects on cancer invasion and metastasis [20], which was proved by differentially expressed genes between primary and lung metastases are involved in the Rap1 signaling pathway [21].

HUGs showed enrichment in proteolysis, negative regulation of the canonical Wnt signaling pathway, hormone activity, plasma membrane, and extracellular exosome of cellular components. Invasive migration of tumor cells depends on proteolytic extracellular matrix (ECM) remodeling [22]. The proteolytic procedure is significant in numerous phases of the metastasis. The level of MMPs is upregulated in breast cancer, leading to enhanced proteolysis and consequently tumor metastasis [23]. Wnt/*β*-catenin pathway activity is essential for osteoblast differentiation, and inactivation of Wnt/*β*-catenin pathway activity takes a vital role in carcinogenesis of osteosarcoma [24]. Studies have shown that tumor exosomes promote the progression of cancer and act as correspondents in the interreaction of tumors and bone cells in skeletal microenvironment [25].

Numerous studies have concluded that the specific diagnosis and assessment of prognosis can be made from the perspective of DNA methylation [9]. Therefore, three aberrant methylation-driven genes (COL13A1, MXI1, and TBRG1) were screened for survival by univariate Cox regression, Venn analysis, and LASSO Cox regression. Risk models were constructed using gene expression levels and Cox regression coefficients. Survival analysis of risk scores showed that patients with high risk scores had poorer survival. The AUC of the risk model in the ROC curve was greater than 0.810. This is the first report on three aberrant methylation-driven gene risk models for OS, which may be a novel prognostic biomarker for OS.

Three genes (COL13A1, MXI1, and TBRG1) were hypermethylated and downregulated in OS. Type XIII collagen is a transmembrane protein located at cell-cell and cell-ECM junctions [26]. This type of collagen is poorly studied in the field of oncology. However, it has been reported that in urothelial carcinoma, the production of collagen type 13 alpha 1 (COL13A1) by tumor cells is closely related to tumor invasion. And studies have shown that urinary COL13A1 protein content can act as an independent risk factor for bladder cancer recurrence. COL13A1 in urine may be a potential diagnostic and prognostic biomarker for bladder cancer. Knockdown of COL13A1 with siRNA resulted in dramatic changes in invasion patterns and reduced invasive capacity by reducing invadopodia [27].

MAX interactor 1 (MXI1) is considered a tumor suppressor gene located in the cancer hotspot region 10q24-q25 of the human chromosome [28]. MXI1 protein specifically competes with MYC for MAX protein [29], preventing the formation of MYC-MAX heterodimers to antagonize the transcriptional activity of MYC [30], and participates in multiple biological processes consist of cell growth and differentiation [31], cell cycle regulation [32], apoptosis, and radiosensitivity [33]. Infection of cultured DU145 prostate cancer cells with adenovirus expressing MXI1 resulted in decreased cell proliferation and a higher proportion of cells in the G [2]/M phase of the cell cycle, and decreased c-MYC expression by MXI1 resulted in cell growth arrest [34]. Loss of the MXI1 allele is found in approximately 50% of melanoma cases and occurs more frequently in recurrent or metastatic tumors [35]. *Mxi1* knockout mice are prone to squamous cell carcinoma and malignant lymphoma, and higher MXI1 protein levels are associated with better breast cancer prognosis [36]. It is suggested that downregulation of MXI1 may contribute to tumorigenesis and correlate with the prognosis of tumor patients [37].

Transforming growth factor beta regulator 1 (TBRG1) acts as a growth inhibitor and tumor suppressor. TBRG1 can activate p53/TP53, causes G1 arrest, and collaborates with CDKN2A to restrict proliferation [38] but does not require either protein to inhibit DNA synthesis. TBRG1 can redistribute CDKN2A into the nucleoplasm and is involved in maintaining chromosomal stability [39]. Inhibition of miR-155 target gene TBRG1 expression by overexpression of miR-155 increased cell proliferation in B-cell lymphomas [40]. Interventions for specific genes need to be analyzed together with the HR value of survival analysis. Thus, promoting the expression of these genes by reducing the DNA methylation levels of these genes can inhibit osteosarcoma development.

Due to the lack of relevant data, the effect of abnormal methylation on gene expression needs to be confirmed by collecting clinical samples. In addition, effects of DNA methylation inhibitors on osteosarcoma cell phenotype and prognosis in animal models targeting the three identified genes (COL13A1, MXI1, and TBRG1) deserve further study.

## 5. Conclusion

After the combined analysis of GSE36002, GSE12865, and TARGET-OS, we discovered the key genes in osteosarcoma that are regulated by DNA methylation and thus affect survival time. Three genes, including *COL13A1, MXI1*, and*TBRG1,* could be used as DNA methylation biomarkers of accurate diagnosis and therapy for osteosarcoma in the future.

## Data Availability

The data used to support the findings of this study are included within the article.

## Supplementary Materials

Supplementary Materials Figure S1: the univariate Cox regression analysis of TARGET-OS project in TCGA. A: forest plot of the top 10 genes of 638 survival-related genes (*P* <0.01). B: Venn analysis of 1318 aberrant methylation-driven genes and 638 survival-related genes.
