# Supplementary figures and images for: Development of a Risk Score Model for Osteosarcoma Based on DNA Methylation-Driven Differentially Expressed Genes
Source: J Oncol. 2022 May 13;2022:7596122. doi: 10.1155/2022/7596122 (PMC9122702; doi:10.1155/2022/7596122)

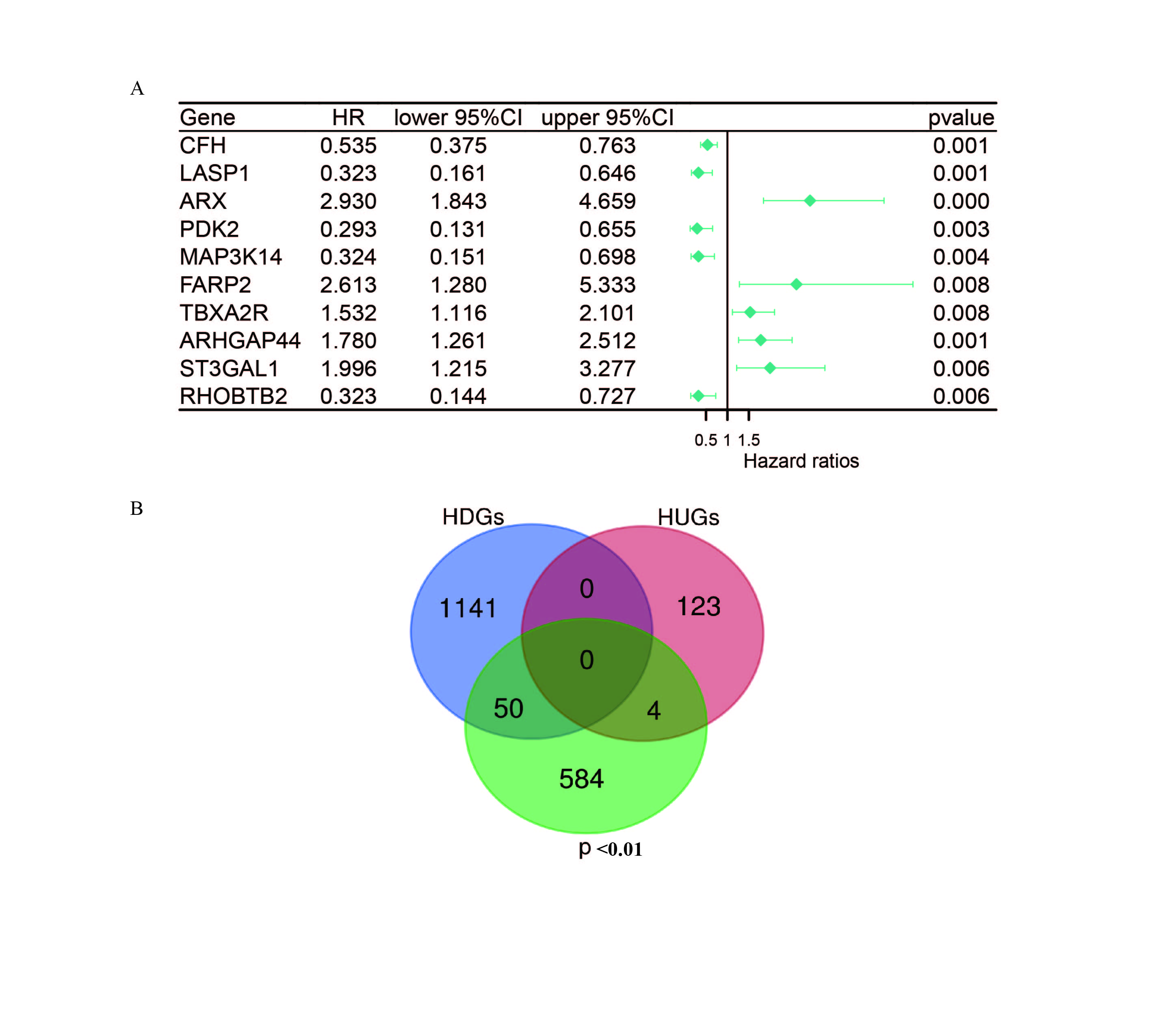

Supplement: Supplementary Materials — Figure S1: the univariate Cox regression analysis of TARGET-OS project in TCGA. A: forest plot of the top 10 genes of 638 survival-related genes (P <0.01). B: Venn analysis of 1318 aberrant methylation-driven genes and 638 survival-related genes. [file 7596122.f1.jpg]
